# Opportunities for Antibody Discovery Using Human Pluripotent Stem Cells: Conservation of Oncofetal Targets

**DOI:** 10.3390/ijms20225752

**Published:** 2019-11-15

**Authors:** Heng Liang Tan, Andre Choo

**Affiliations:** 1 Bioprocessing Technology Institute, Agency for Science, Technology and Research (A*STAR), Biopolis, Singapore 138668, Singapore; andre_choo@bti.a-star.edu.sg; 2 Department of Biochemical Engineering, National University of Singapore, Singapore 117575, Singapore

**Keywords:** pluripotent stem cells, antibody discovery, regenerative cell therapy, oncology, oncofetal

## Abstract

Pluripotent stem cells (PSCs) comprise both embryonic stem cells (ESCs) and induced pluripotent stem cells (iPSCs). The application of pluripotent stem cells is divided into four main areas, namely: (i) regenerative therapy, (ii) the study and understanding of developmental biology, (iii) drug screening and toxicology and (iv) disease modeling. In this review, we describe a new opportunity for PSCs, the discovery of new biomarkers and generating antibodies against these biomarkers. PSCs are good sources of immunogen for raising monoclonal antibodies (mAbs) because of the conservation of oncofetal antigens between PSCs and cancer cells. Hence mAbs generated using PSCs can potentially be applied in two different fields. First, these mAbs can be used in regenerative cell therapy to characterize the PSCs. In addition, the mAbs can be used to separate or eliminate contaminating or residual undifferentiated PSCs from the differentiated cell product. This step is critical as undifferentiated PSCs can form teratomas in vivo. The mAbs generated against PSCs can also be used in the field of oncology. Here, novel targets can be identified and the mAbs developed as targeted therapy to kill the cancer cells. Conversely, as new and novel oncofetal biomarkers are discovered on PSCs, cancer mAbs that are already approved by the FDA can be repurposed for regenerative medicine, thus expediting the route to the clinics.

## 1. Introduction

Pluripotent stem cells (PSCs) comprise both embryonic stem cells (ESCs) and induced pluripotent stem cells (iPSCs) [1,2]. The former is derived from the inner cell mass of the blastocyst while the latter is generated by reprogramming somatic cells through the introduction of pluripotent-inducing transcription factors [3,4,5,6]. Both types of PSCs exhibit similar properties of self-renewal and the ability to differentiate into cell types representing the three germ layers. Hence, PSCs are widely accepted as an inexhaustible source of cells for various applications and are prominent in the area of regenerative cell therapy. Many studies have investigated the differentiation of PSCs for the treatment of injuries and degenerative diseases such as diabetes, spinal cord injury, muscular dystrophy, cardiac and liver failure to list a few examples [7,8,9,10,11,12]. Studies have shown that PSCs can also be differentiated to innate immune cells such as natural killer cells and T-cells for cancer therapies [13,14]. The field of PSCs is advancing rapidly as evident from the significant increase of published research papers and the number of clinical trials with PSC derivatives, especially for the treatment of macular degeneration [15,16].

To meet clinical standards and demands, the bioprocessing of PSCs is necessary and is analogous to that of manufacturing a biologics drug. A typical bioprocess for cell therapy starts from the “raw material” of undifferentiated PSCs, expansion, differentiation, harvesting, isolation and validation of final product prior to delivery to the clinics [17,18]. At every stage of the bioprocess, the cells have to be checked for quality, and one way of carrying the quality checks is through the use of monoclonal antibodies (mAbs). In addition, mAbs can also be used for isolation of the cellular products.

During the early stage of human ESC (hESC) research, the availability of biomarkers used to characterize hESCs was limited. A list of these biomarkers used for PSCs is summarized by Carpenter et al. [19]. Many of these biomarkers were originally discovered in other cell types such as embryonal carcinoma (EC) or teratocarcinoma cells, affirming the presence of oncofetal antigens on PSCs [20]. For example, the surface antigens (stage-specific embryonic antigen) SSEA-3 and SSEA-4, together with TRA-1-60 and TRA-1-81 were found to be expressed on ECs [21,22,23,24,25]. Hence, various groups proceeded to raise antibodies specific to surface biomarkers on hESCs with the primary objective of characterizing and validating the PSCs and to use these mAbs to “clean up” the final cell product for regenerative cell therapy. The conservation of some oncofetal antigens between embryonic cells and cancer cells also provided an opportunity to discover novel mAbs to be used in the field of targeted therapy for cancers. These mAbs can then be engineered to a variety of different formats to target and kill cancer cells, e.g., naked mAbs (antibody-dependent cell-mediated cytotoxicity, ADCC, or enhanced ADCC), antibody drug conjugate (ADC) or bispecific antibodies.

## 2. mAbs for Regenerative Cell Therapy

The mAbs that were originally raised against ECs or teratocarcinoma cells served well as biomarkers for PSCs. However, groups continued to generate mAbs against hESCs with the objectives of discovering novel biomarkers specific to PSCs and using these mAbs to characterize PSCs better. To achieve this, PSCs were used as immunogens. It was not surprising to note that the antigen targets of these mAbs can also be found on cancer, thus confirming the conservation of oncofetal targets between PSCs and cancers. In this section, we will give a review of some mAbs that were raised using PSCs as immunogens for the application in regenerative cell therapy.

Choi et al. raised a panel of 33 mAbs specific to undifferentiated hESCs using a modified decoy immunization strategy, whereby mice were immunized with hESCs as the main immunogen and differentiated hESCs (in the presence of retinoic acid) [26,27]. Characterization of one of the mAbs identified the antigen target as CD9 [26]. They also identified two other mAbs that bind to surface proteins on both hESCs and cancer cell lines, though the identities of the antigens were not determined [27]. Independently, Son et al., using conventional hybridoma technology, raised a panel of mAbs against hESC, of which two mAbs were shortlisted and characterized [28,29]. The first mAb, 4-63, was found to bind to the antigen L1 cell adhesion molecule (L1CAM) on undifferentiated PSCs [28]. L1CAM was found to play an important role in self-renewal by activating fibroblast growth factor receptor 1 signaling. The second mAb binds to desmoglein-2 (DSG2) on undifferentiated hESCs, and its surface expression is rapidly downregulated upon differentiation [29]. Their studies further revealed that DSG2 is essential to the self-renewal of PSCs and the acquisition of pluripotency during somatic cell reprogramming by controlling β-catenin/slug-mediated epithelial mesenchymal transition. In another study, a panel of mAbs to cell surface antigens on hESCs was also generated [30]. Characterization of the mAb, 20-202S, showed that the antigen target was heat shock 70 kDa protein 8 isoform 1 (HSPA8), which is downregulated upon differentiation. Additionally, HSPA8 protein belongs to the family of heat shock protein 70, which is found in some cancers. Through western blotting, the mAb 20-202S was shown to bind to cancer cells (cholangiocarcinoma, sarcomatoid cholangiocarcinoma and cervical cancer) that expressed this oncofetal antigen.

Our group has also generated panels of mAbs to hESCs [31,32,33,34,35]. Taking a whole cell immunization approach, mAbs to podocalyxin-like protein 1 (PODXL), epithelial cell adhesion molecule (EpCAM), annexin A2 and Erbb-2 were obtained. These mAbs bind to either protein targets or glycan epitopes [36,37]. In addition to characterizing PSCs, we demonstrated the utilities of our mAbs in both regenerative cell therapy and oncology. In regenerative cell therapy, the mAbs can be used to purify the final cell product by removing undifferentiated PSCs through cell separation methods and cytotoxic mAbs specific to undifferentiated PSCs [31,32,36,38]. Some of the mAbs also bind to cancer cells as a result of oncofetal antigen conservation and have been developed as targeted therapies to treat cancers [34,35].

Besides their use to characterize PSCs, mAbs can also be used to separate undifferentiated cells from the differentiated cell product, especially if the antigen is downregulated in the latter. One of the safety concerns associated with the use of PSCs is the presence of residual undifferentiated PSCs in the differentiated cell product. The contaminating undifferentiated PSCs can potentially form teratomas in vivo [31,32,38,39,40]. Hentze et al. demonstrated that 245 undifferentiated hESCs were all that was needed to form teratomas in SCID mice [41]. Fujikawa et al. showed that insulin-expressing cells derived from ES cells, post-transplanted into SCID mice, were able to form teratomas in vivo resulting in the failure of treatment for type I diabetes [42]. In another case study, researchers from Israel reported that four years after a patient was given fetal neural stem cell transplants to treat ataxia telangiectasia, the patient developed tumors in his brain and spinal cord. This case study highlights the risk of teratoma or tumor formation not just from pluripotent stem cells but also from other sources of stem cells (including fetal neural stem cells) and hence, poses a major stumbling block for cell-based therapies [43,44].

*In lieu* of this safety concern, Kornelia et al. used mAbs that were raised against hESCs, coupled with magnetic activated cell sorting (MACS), to separate cell mixtures of undifferentiated hESCs and fibroblasts [38]. Validating the final separated product via phenotype (flow cytometry) and genomic (quantitative reverse transcription polymerase chain reaction, RT-qPCR) analysis, they were able to remove 97.2–99.7% of undifferentiated hESCs from the cell mixture. When the enriched fibroblast cells (after the one-step MACS) were transplanted into severe combined immunodeficiency (SCID) mice, 8 out of the 9 mice did not develop teratomas while the teratoma formation in the last mouse was significantly delayed. They further demonstrated that by selectively removing undifferentiated hESCs using MACS followed by treatment with a cytotoxic antibody (mAb 84) specific to undifferentiated hESCs, they were able to remove 99.1–100% of undifferentiated hESCs from the cell mixture [31,32,38].

The cytotoxic mAb specific to undifferentiated hESCs, mAb 84, that Kornelia et al. used is an IgM. The antigen target of mAb 84 was found to be podocalyxin-like protein 1 (PODXL) [31]. The calculated molecular weight of PODXL is 55 kDa but the apparent mass in non-ES cells is approximately 160 kDa as the protein is highly glycosylated [45]. PODXL is reported to be a biomarker of hESCs, and a study by Schopperle and DeWolf confirmed the presence of a stem cell PODXL with a molecular weight of 200 kDa [46,47]. mAb 84 kills PSCs rapidly via oncosis, by forming pores on the plasma membrane, probably because of antigen aggregation by the IgM [32]. When hESCs were pre-treated with mAb 84 and injected into SCID mice, they were able to prevent the formation of teratomas even up to 20 weeks and consequently, enhance the safety of PSC regenerative therapy.

Matsumoto et al. reported another cytotoxic mAb which was generated using iPSCs as the immunogen [48]. The mAb, R-17F, is an IgG1 and was found to specifically bind to PSCs but not to ECs. R-17F kills PSCs in a dose-dependent manner and its cytotoxicity was significantly enhanced through hyper-crosslinking with a secondary antibody. Unlike mAb 84, R-17F does not bind to a glycoprotein and its epitope was identified as the glycolipid lacto-*N*-fucopentose I (LNFP I).

Two other mAbs, A1 and mAb-A4, have been reported to kill undifferentiated PSCs and bind to glycan epitopes [36,37]. mAb A1, is an IgG and the mechanism with which A1 kills PSCs was also elucidated to be oncosis. Unlike the IgM mAb 84, oncosis by A1 is mediated by excess reactive oxygen species production [36]. Another antibody, mAb-A4, an IgM, was also found to kill PSCs rapidly [37]. Interestingly, both mAbs recognized glycan epitopes expressed on multiple proteins. Via glycan inhibition assays, A1 was found to bind to glycans containing the motif Fuc*α*1-2Gal*β*1-3GlcNAc*β*1-3Gal*β*1. Using a combination of techniques and assays (enzymatic digestion with glycosidases, glycan microarrays, siRNA and high sensitivity matrix-assisted laser desorption/ionization mass spectrometry), the terminal epitopes of mAb-A4 were found to be type 1 LacNAc and H type 1 sugars. Tang et al. also reported of a mAb, SSEA-5, which binds to the glycan H type 1 that is highly and specifically expressed in PSCs [49]. In their study, they showed that a combination of SSEA-5 with two other pluripotent surface markers is sufficient to remove teratoma formation potential of PSCs.

Recently, our group proposed a strategy to complement existing methods that eliminate teratoma-forming cells in vitro [50]. Residual undifferentiated PSCs could possibly escape in vitro removal methods and be introduced into patients together with the differentiated cells. Here, we demonstrated that mAbs, which elicit antibody-dependent cell-mediated cytotoxicity (ADCC) or as an antibody drug conjugate (ADC), can be included as an additional safeguard to eliminate these “escaped” undifferentiated cells that are circulating in the body and consequently enhance the safety of PSCs-derived cell therapies. As a proof of concept, mAb 2448, which targets annexin A2 on PSCs, was able to eliminate hESCs in vivo via both ADCC and ADC mechanisms of action. Interestingly, the ADC when administered at a site away from the cell transplant was still able to home towards the circulating undifferentiated PSCs and prevent or delay teratoma formation.

Hence, in the process of raising antibodies to surface proteins of PSCs, novel biomarkers were discovered and can be used to characterize the undifferentiated PSCs. Table 1 provides a summary of the mAbs highlighted in this review section. These biomarkers can be either protein or glycan in nature. In addition to using them to characterize PSCs, these mAbs can also be used in combination to separate and ”clean up” the undifferentiated PSCs from final cell products, prevent teratoma formation in vivo and consequently make PSC-derived regenerative cell therapies safer.

## 3. mAbs for Oncology

From the list of mAbs in Table 1, it is evident that many of the antigen targets identified on PSCs are also expressed on cancer cells [51,52,53,54,55,56,57]. This is not surprising as there are many studies that support the conservation of antigens between embryonic cells and cancer cells [58,59,60,61,62]. Historically, fetal and embryonic materials have also been investigated and used as alternatives for cancer treatment. Schöne found that immunization of mice with fetal material resulted in the rejection of transplanted tumors. Fibiger and Moeller extended this study and showed that immunization of fetal skin into mice prevented the growth and metastasis of coal tar-induced carcinoma [58]. In recent studies, immunization of mice with either human fetal tissues or PSCs showed strong protection against cancer tumor establishment and proliferation [59,60]. Antigens associated with embryonic and fetal development, which are also found in cancers, are classified as oncofetal antigens. For example, the surface markers TRA-1-60 and the SSEA-3, originally raised against ECs, were also identified in breast and prostate cancer subpopulations [61]. Some other common oncofetal antigens that are used as biomarkers in oncology include cancer antigen 125 (CA125 or Mucin16) for ovarian cancer, the sialylated Lewis A antigen CA19-9 for pancreatic cancer, α-fetoprotein (AFP) for hepatocellular cancer and germ cell tumors and prostate-specific antigen (PSA) [62,63,64]. As described earlier, mAb 84 binds to PODXL on hESCs [31,32]. PODXL is reported to be expressed in multiple cancer types such as breast, esophageal, colorectal cancers, lung and gastric adenocarcinoma, pancreatic and urothelial bladder cancers [65,66,67,68,69,70,71,72,73]. Besides their applications in PSC-derived regenerative cell therapies, mAbs against PSCs also provide the opportunity to discover novel mAbs and new biomarkers against cancers. For example, Kim et al., Son et al. and Choo et al. (Table 1) reported that their mAbs raised against hESCs also bind to cancer cells [27,30,37].

When mAbs bind to the targeted cancer cells, they are able to kill the cells through various mechanisms of action (MOAs). The Fc-region of antibodies plays an important role in the activation of immune cells and the killing of targeted cells via ADCC and also in mediating cell killing through complement-mediated cytotoxicity (CDC) [74,75,76,77,78]. Antibodies can also cause vascular and stromal cell ablation, affecting cancer cell proliferation. Alternatively, antibodies can neutralize or block the binding of growth factors to the respective receptors and consequently inhibit cell proliferation [74,75,76,77]. They can also mediate direct cell killing by the activation of apoptotic pathways or via oncosis [75,76,78,79,80,81]. Antibodies can also internalize into the targeted cells and deliver payloads, such as drugs, cytotoxic or radiation agents, to directly kill the cancer cells [74,75,76,78]. Hence, oncofetal antigens are promising targets for antibody-based therapies against cancers. In this section, we will highlight some mAbs that were generated against hESCs. These hESC mAbs also bound to various cancers, and some were able to kill the cancer cells via various MOAs and cause tumor regression in vivo.

Ng et al. reported a mAb raised against hESCs [33]. Through immuno-precipitation and mass spectrometry, the antigen target of this mAb (mAb 8) was found to be epithelial cell adhesion molecule (EpCAM). This biomarker is shown to be a surface marker on undifferentiated hESCs and the expression of EpCAM is downregulated upon differentiation. Knockdown and silencing of EpCAM via small interfering ribonucleic acid (siRNA) and short hair RNA (shRNA) had a marginal effect on the expression of other pluripotent markers (OCT-4, TRA-1-60 and NANOG) in hESCs but decreased the proliferation of hESCs significantly. Interestingly, teratoma formation was comparable between the EpCAM shRNA cells and control. However, through gene expression analysis of the teratomas, the EpCAM shRNA samples demonstrated the greatest significant increase in the endoderm marker AFP. EpCAM is reported to be highly expressed in epithelial carcinomas and also expressed in numerous cancers such as ovarian, breast, colorectal adenocarcinomas and gastric cancers [82,83,84,85,86,87,88]. This is consistent with our observation that mAb 8 also binds to various cancer cell lines, including breast and ovarian cancers.

Another hESC mAb, 2448, binds to annexin A2 on hESCs and on cancer cells [34,50]. Cua et al. showed that the mAb binds to glycans on annexin A2, which confers the mAb a unique property. The mAb binds specifically to cancer cells with epithelial phenotype. Upon binding to ovarian and breast cancer cells, 2448 is able to internalize into the cells and when developed as an ADC, the mAb was able to kill the cancer cells in vitro. The mAb was also chimerized from a mouse IgG with a human IgG1 backbone. The chimerized 2448 was able to elicit ADCC in vitro, and further engineering of the chimeric mAb to remove fucose in the Fc domain enhanced the ADCC more than 20-fold. in vivo, the chimerized mAb was able to home in to the tumors and cause tumor regression in ovarian xenograft models [34].

In a study by Tan et al., the mAb, A19, bound to breast and ovarian cancer cells [35]. The antigen target of A19 was identified as Erbb-2. Errb-2 is highly expressed on hESCs and many cancers and plays an important role in cell proliferation [89,90]. This antigen serves as a good target for Herceptin, which is the gold standard for targeted therapy in the clinics to treat HER2 positive breast cancers and gastric cancers [91,92,93]. Tan et al. also demonstrated that the epitope of the A19 is N-glycan on the protein and binds to isoforms different to Herceptin [35]. Unlike 2448, the chimeric A19 does not elicit ADCC but, when developed as an ADC, was able to kill the cancer cells in vitro. In xenograft models, under sub-optimal conditions, the ADC was able to reduce the volume of the tumors by 60% compared to the controls.

The conservation of oncofetal targets (proteins or glycans) enabled mAbs that were originally generated against PSCs to be repurposed for targeted therapy in cancers (Table 2). mAbs have been a success as targeted therapies in the clinics and are able to elicit various MOAs to kill cancer cells. However, a major challenge is finding new biomarkers to target the cancer cells. Furthermore, many oncology mAbs are raised towards a specific disease indication. Hence, anti-PSC mAbs provide the opportunities for discovery of new biomarkers in a non-biased manner, and these mAbs can be repurposed for oncology.

## 4. Discussion

In this review, we summarized some mAbs that were generated following mice immunization with PSCs. Subsequently, these mAbs were also found to bind cancer cells due to the conservation of oncofetal targets between embryonic and cancer cells. These studies also provided the opportunities for the identification of novel conserved antigens, and interestingly, the majority of the antigen targets, which are expressed on both PSCs and cancer cells, are glycoproteins.

It is to be noted that the expression of oncofetal antigens is not limited to PSCs. Studies have also shown that these antigens can also be expressed on fetal cells, which may also be used as immunogens to raise mAbs against cancers [94]. At the same time, some of these oncofetal antigens are biomarkers in both cancers and differentiated PSCs. For example, α-fetoprotein (AFP) and carcinoembryonic antigen (CEA) are expressed in cancers but also upregulated in PSC-derived hepatocytes and dendritic cells, respectively [95,96,97,98,99,100,101,102]. However, a distinguishing feature of cancer cells/PSCs from normal/differentiated cells could be the post-translational modifications (e.g., glycans) of these antigens, which serve as an additional layer of resolution between the cell types and cell stages [103,104]. An example of this was reported previously by Schopperle and DeWolf [47]. They identified the presence of a stem-cell-specific PODXL on PSCs, which was different from non-ES cells due to differences in the glycan profile [47].

In this review, we focus on mAbs raised against PSCs. This duality in binding enables the mAbs to be used for both stem cell and cancer cell applications. In regenerative cell therapy, the mAbs can be used to characterize the PSCs. The mAbs can also be used for cell separation and to remove contaminating or residual undifferentiated PSCs from the final differentiated cell product. This step is critical in making PSC regenerative therapy safer as undifferentiated PSCs can form teratomas in vivo. Due to the conservation of oncofetal targets between embryonic and cancer cells, many of the mAbs against PSCs bind to cancer cells. For oncology applications, these mAbs can then be engineered to a variety of different formats to target and kill cancer cells, e.g., naked mAbs (ADCC or enhanced ADCC), ADC or bispecific antibodies.

The biomarkers identified in Table 1 and Table 2 reinforce the concept of oncofetal antigen conservation between embryonic stem cells and cancer cells. As mentioned, oncology mAbs are usually raised towards specific disease indications. PSCs can be used as unbiased sources to generate mAbs for oncology applications and treating various types of cancers. Conversely, there are reports of mAbs that are used as targeted therapy to treat cancers and have been found to bind to undifferentiated PSCs. Sougawa et al. demonstrated that the FDA-approved ADC, brentuximab vedontin, is able to eliminate undifferentiated CD30-positive human induced pluripotent stem cells (hiPSCs) during cardiomyocyte differentiation and prevent teratoma formation [105].

In conclusion, mAbs against PSCs have dual use in the fields of regenerative medicine and targeted therapy for oncology. The many FDA-approved mAbs, which were developed for cancer therapies, can also be explored and used to characterize PSCs, thereby making regenerative therapies safer and expediting the development route of these mAbs to the clinics.

## Figures and Tables

**Table 1:** Summary of mAbs generated against PSCs and used for regenerative cell therapy.

mAbs	Immunogen	Antigen Target	Antigen Type ^1^	Cells that mAbs Bind ^1^	Authors (References)
L125-C2	hESC	CD9	Protein	PSC	Choi et al. [26]
63-B6	hESC	ND ^2^	Protein	PSC, EC, Cancers	Kim et al. [27]
246-D7	hESC	ND ^2^	Protein	PSC, EC, Cancers	Kim et al. [27]
4-63	hESC	L1CAM	Protein	PSC	Son et al. [28]
K6-1	hESC	DSG2	Protein	PSC	Park et al. [29]
20-202S	hESC	HSPA8	Protein	PSC, Cancers	Son et al. [30]
mAb 84	hESC	PODXL	Glycoprotein	PSC, EC	Choo. et al. Tan et al. [31,32]
R-17F	iPSC	Lacto-*N*-fucopentose I	Glycolipid	PSC	Matsumoto. et al. [48]
A1	hESC	Fuc*α*1-2Gal*β*1-3GlcNAc*β*1-3Gal*β*1	Glycan	PSC	Zheng et al. (36)
mAb-A4	hESC	Type 1 LacNAc and H Type 1	Glycan	PSC, Cancers	Choo. et al. [37]
SSEA-5	hESC	H Type 1	Glycan	PSC	Tang. et al. [49]
2448	hESC	Annexin A2	Glycoprotein	PSC, Cancers	Tan et al. [50]

^1^ As reported by authors; ^2^ ND: Not determined; EC, embryonal carcinoma; PSCs include either human embryonic stem cells (hESCs)/induced pluripotent stem cells (iPSCs) or both in this table.

**Table 2:** Summary of mAbs generated against PSCs and used in oncology.

mAbs	Immunogen	Antigen Target	Antigen Type ^1^	Cells that mAbs Bind ^1^	Mechanism of Action (MOA)	Authors (References)
mAb 8	hESC	EpCAM	Protein	PSC, Cancers	No MOA	Ng et al. [33]
2448	hESC	Annexin A2	Glycoprotein	PSC, Cancers	Internalization (ADC), ADCC	Cua et al. [34]
A19	hESC	Erbb-2	Glycoprotein	PSC, Cancers	Internalization (ADC)	Tan et al. [35]

^1^ As reported by authors; PSCs include either hESCs/iPSCs or both in this table; ADC, antibody drug conjugate; ADCC, antibody-dependent cell-mediated cytotoxicity; EPCAM, epithelial cell adhesion molecule.
